# Exploring Potential Biomarkers and Molecular Mechanisms of Ischemic Cardiomyopathy and COVID-19 Comorbidity Based on Bioinformatics and Systems Biology

**DOI:** 10.3390/ijms24076511

**Published:** 2023-03-30

**Authors:** Simin Luo, Xuan Zhang, Xiang Xiao, Wenting Luo, Zixuan Yang, Songqi Tang, Wei Huang

**Affiliations:** 1 School of Basic Medical Sciences, Chengdu University of Traditional Chinese Medicine, Chengdu 611137, China; 2 College of Traditional Chinese Medicine, Hainan Medical University, Haikou 571199, China

**Keywords:** bioinformatics, ischemic cardiomyopathy, COVID-19, pathogenesis, disease markers

## Abstract

Cardiovascular complications combined with COVID-19 (SARS-CoV-2) lead to a poor prognosis in patients. The common pathogenesis of ischemic cardiomyopathy (ICM) and COVID-19 is still unclear. Here, we explored potential molecular mechanisms and biomarkers for ICM and COVID-19. Common differentially expressed genes (DEGs) of ICM (GSE5406) and COVID-19 (GSE164805) were identified using GEO2R. We performed enrichment and protein–protein interaction analyses and screened key genes. To confirm the diagnostic performance for these hub genes, we used external datasets (GSE116250 and GSE211979) and plotted ROC curves. Transcription factor and microRNA regulatory networks were constructed for the validated hub genes. Finally, drug prediction and molecular docking validation were performed using cMAP. We identified 81 common DEGs, many of which were enriched in terms of their relation to angiogenesis. Three DEGs were identified as key hub genes (*HSP90AA1*, *HSPA9*, and *SRSF1*) in the protein–protein interaction analysis. These hub genes had high diagnostic performance in the four datasets (AUC > 0.7). Mir-16-5p and KLF9 transcription factor co-regulated these hub genes. The drugs vindesine and ON-01910 showed good binding performance to the hub genes. We identified *HSP90AA1*, *HSPA9*, and *SRSF1* as markers for the co-pathogenesis of ICM and COVID-19, and showed that co-pathogenesis of ICM and COVID-19 may be related to angiogenesis. Vindesine and ON-01910 were predicted as potential therapeutic agents. Our findings will contribute to a deeper understanding of the comorbidity of ICM with COVID-19.

## 1. Introduction

COVID-19 (SARS-CoV-2) is currently wreaking havoc around the world. Lung disease, the most common complication of COVID-19, can present as a progression from an asymptomatic/mild infection to multi-organ failure leading to death [1,2,3]. Besides lung disease, cardiovascular complications are also clinically recognized as risk factors for COVID-19 [4,5]. Approximately 20–30% of patients with COVID-19 who show cardiovascular manifestations have poorer outcomes [6,7]. In China, a study of 44,672 COVID-19 cases found that the death rate from combined cardiovascular complications was 10.5% compared with an overall rate of 2.4% [8]. Cardiovascular complications can be triggered by myocardial ischemia or non-ischemic processes (e.g., myocarditis) [9], and may be related to viral damage and immune responses [10,11,12].

Ischemic cardiomyopathy (ICM) reduces or stops blood flow to the heart, leading to myocardial damage [13]. In a systematic review, Omidi et al. [14] found that cardiomyopathy was a very common cardiovascular complication of COVID-19. The autopsy pathology of patients with COVID-19 found microscopic evidence that suggested acute ischemic injury [15]. Impaired cardiac systolic function was detected in patients with COVID-19 and ICM by cardiac computed tomography [16]. However, the common pathogenesis of ICM and COVID-19 remains to be fully elucidated.

**Figure 1:**
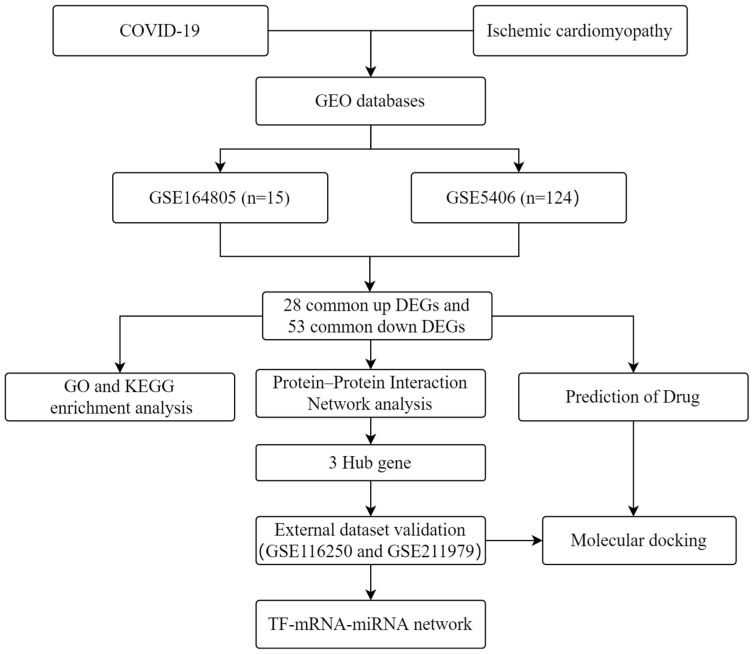
Workflow used in this study to explore potential biomarkers and molecular mechanisms of COVID-19 and ischemic cardiomyopathy by bioinformatics and systems biology methods.

Disease–disease relationships play important roles in the pathobiological manifestations of diseases and in their precise treatment [17]. Exploring disease associations can enhance the understanding of connections between diseases, which can help in developing diagnostic, prognostic, and treatment strategies [18]. Mutations in functionally relevant genes are known to be responsible for overlapping clinical phenotypes of different diseases. Therefore, in this study, we focused on key genes in ICM that are connected to the pathogenesis of COVID-19. The workflow of this study is shown in Figure 1. We analyzed two high-throughput sequencing datasets (GSE164805 and GSE5406) that we downloaded from the Gene Expression Omnibus (GEO). We used an integrated bioinformatics and systems biology approach to identify differentially expressed genes (DEGs) and explored their molecular mechanisms in COVID-19 and ICM. These genes provide an entry point to explore the common clinical phenotypes of COVID-19 and ICM. The protein–protein interaction (PPI) analysis identified key hub genes. To explore the diagnostic performance for these hub genes, we downloaded two other GEO datasets (GSE116250 and GSE211979) and plotted receiver operating characteristic (ROC) curves. Transcription factor (TF) and microRNA (miRNA) regulatory networks for these genes were constructed. We identified potential therapeutic agents by drug prediction, and validated them by molecular docking computer simulations.

## 2. Results

### 2.1. Collection of Common DEGs for COVID-19 and ICM

**Figure 2:**
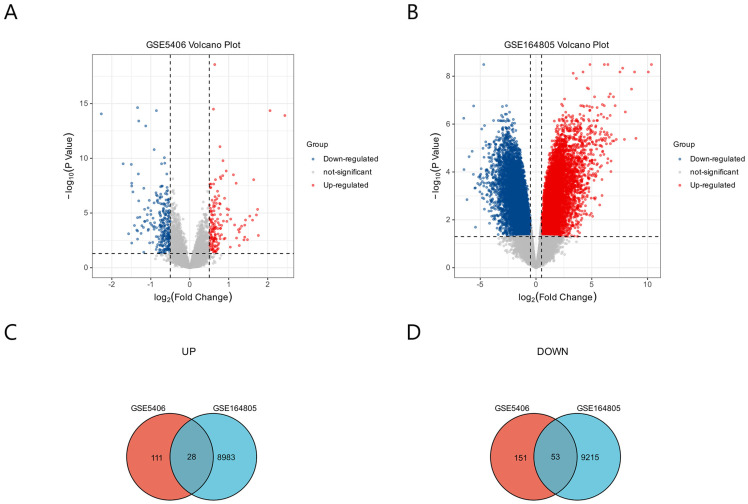
Identification of DEGs common to COVID-19 and ICM using GEO2R. (**A**) DEGs from GSE5406 related to ICM. (**B**) DEGs from GSE164805 related to COVID-19. The *p* values are Bonferroni corrected/adjusted *p* values. (**C**) DEGs co-up-regulated by COVID-19 and ICM. (**D**) DEGs co-down-regulated by COVID-19 and ICM.

In the GSE5406 dataset, 204 genes were up-regulated and 139 genes were down-regulated (Figure 2A). In the GSE164805 dataset, 9268 genes were up-regulated and 9011 genes were down-regulated (Figure 2B). We analyzed the intersection of the DEGs from these two datasets and identified 81 common DEGs; 28 were co-up-regulated and 53 were co-down-regulated for COVID-19 and ICM (Figure 2C,D).

### 2.2. Enrichment Analysis of Common DEGs for COVID-19 and ICM

**Figure 3:**
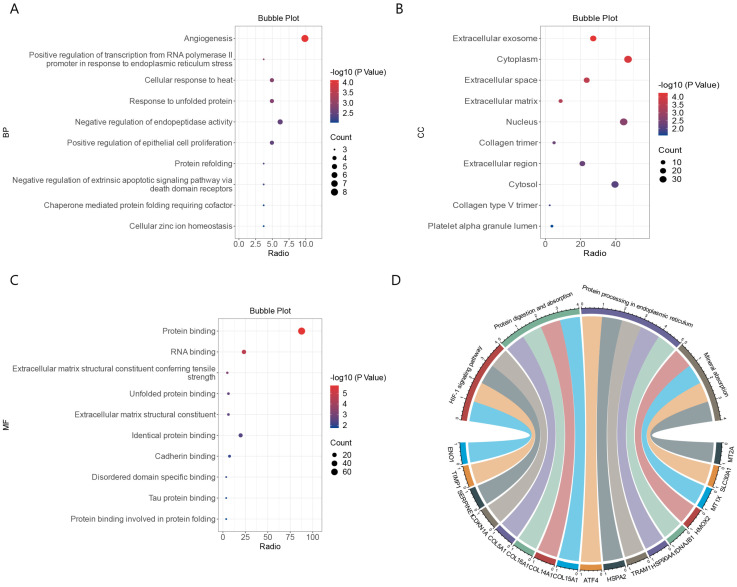
Functional enrichment analysis of DEGs common to COVID-19 and ICM. (**A**) Enriched gene ontology (GO) terms under biological process. (**B**) Enrichment GO terms under cellular component. (**C**) Enriched GO terms under molecular function. (**D**) String diagram of enriched KEGG pathways.

We performed a functional enrichment analysis of the 81 common DEGs using the DAVID database. The DEGs were enriched mainly with the gene ontology (GO) terms angiogenesis under the biological process category, various extracellular components under the cellular component category, and transcriptional regulation of endoplasmic reticulum stress under the molecular function category (Figure 3A–C). The DEGs were enriched mainly with Kyoto Encyclopedia of Genes and Genomes (KEGG) pathways related to mineral absorption and protein processing in endoplasmic reticulum. (Figure 3D). These results strongly suggest that common biological processes and pathways are involved in the development of ICM and COVID-19.

### 2.3. Construction of PPI Networks to Identify Hub Genes

**Figure 4:**
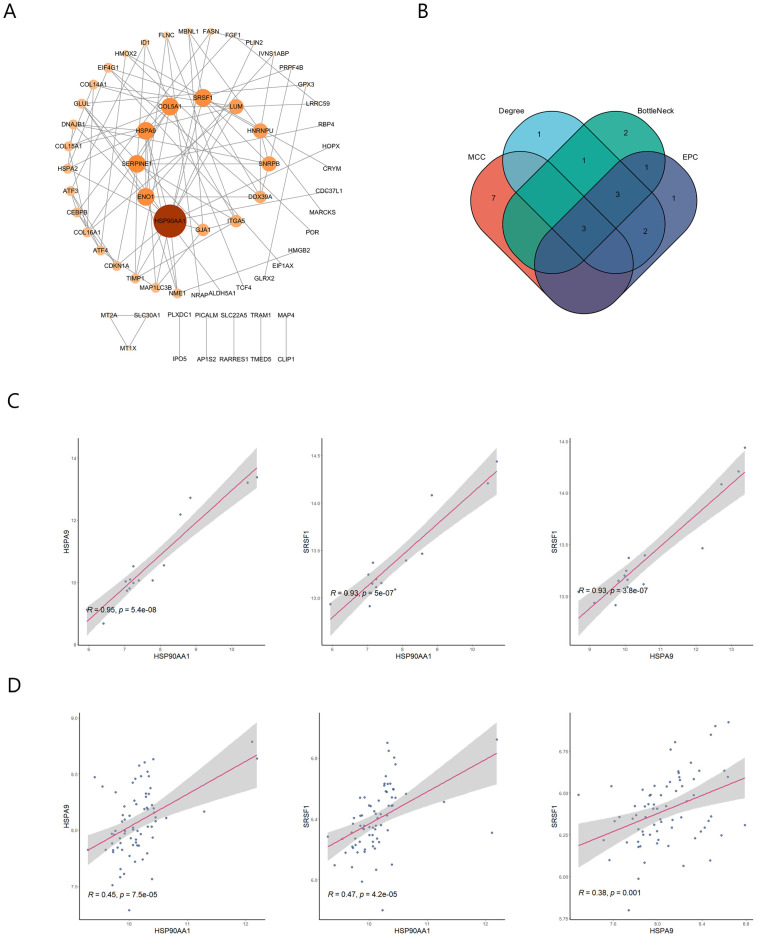
Detection of hub genes by protein–protein interaction network analysis. (**A**) Protein–protein interaction network. The greater the degree of connectivity (degree) between a gene and other genes, the larger the node area and the darker the color. (**B**) Screening of the top 10 hub genes in the network using the MCC, EPC, Degree, and BottleNeck methods in the cytoHubba plugin. (**C**) Correlation of hub genes in the GSE164805 dataset. (**D**) Correlation of hub genes in the GSE5406 dataset.

Individual nodes were hidden to obtain the PPI network. The network was optimized using Cytoscape and 62 nodes were detected (Figure 4A). The top 10 hub genes in the PPI were screened using four methods (MCC, EPC, Degree, BottleNeck) in the cytoHubba plugin (Table 1). The intersection of the results identified three pivotal genes, *HSP90AA1*, *HSPA9*, and *SRSF1* (Figure 4B), for which the expression levels were positively correlated with each other (*p* < 0.01). These three genes may be key genes in the co-morbidity of COVID-19 and ICM.

### 2.4. Validation of Hub Genes Using ROC Curve Plots

**Figure 5:**
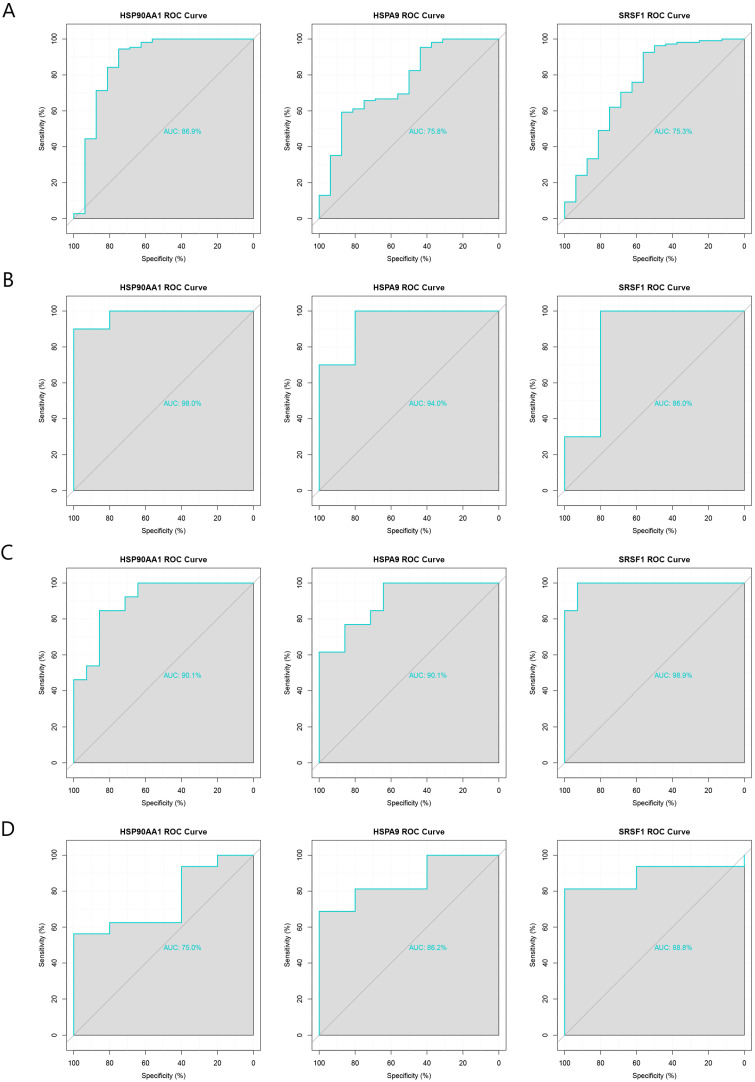
Validation of the diagnostic performance of the three pivotal genes using internal and external datasets. (**A**) ROC curves of the pivotal genes in the GSE5406 dataset (internal). (**B**) ROC curves of the pivotal genes in the GSE164805 dataset (internal). (**C**) ROC curves of the pivotal genes in the GSE116250 dataset (external). (**D**) ROC curves of the pivotal genes in the GSE211979 dataset (external).

GSE5406 and GSE164805 were used as internal datasets, and GSE116250 and GSE211979 were used as external datasets to plot ROC curves for the three hub genes, *HSP90AA1*, *HSPA9*, and *SRSF1*. All three genes had good diagnostic performances with area under the ROC curve (AUC) >70% in both the internal and external datasets (Figure 5A–D). We therefore concluded that *HSP90AA1*, *HSPA9*, and *SRSF1* are pivotal genes in the co-morbidity of COVID-19 and ICM and have considerable diagnostic value.

### 2.5. Construction of TF–mRNA and mRNA–miRNA Regulatory Networks

**Figure 6:**
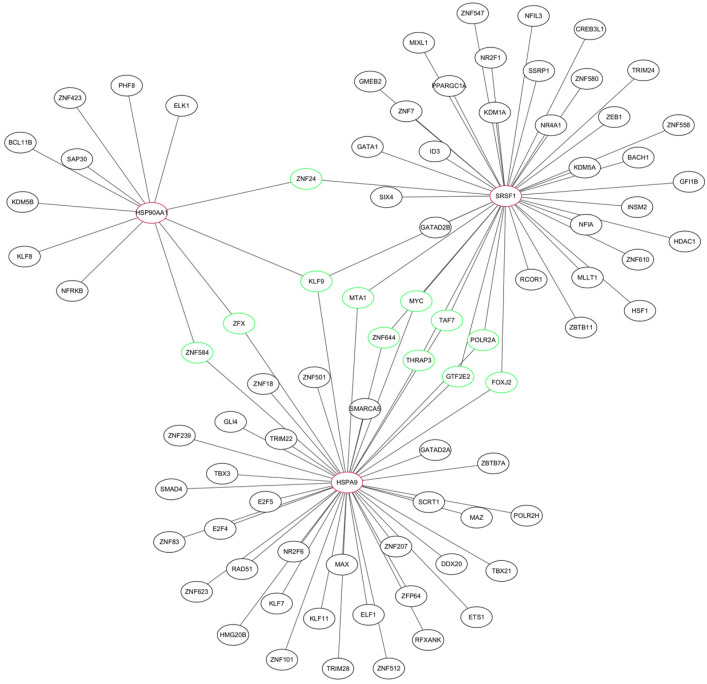
TF–mRNA regulatory network of the three hub genes. Red indicates hub genes, green indicates transcription factors (TFs) associated with at least two of the hub genes.

**Figure 7:**
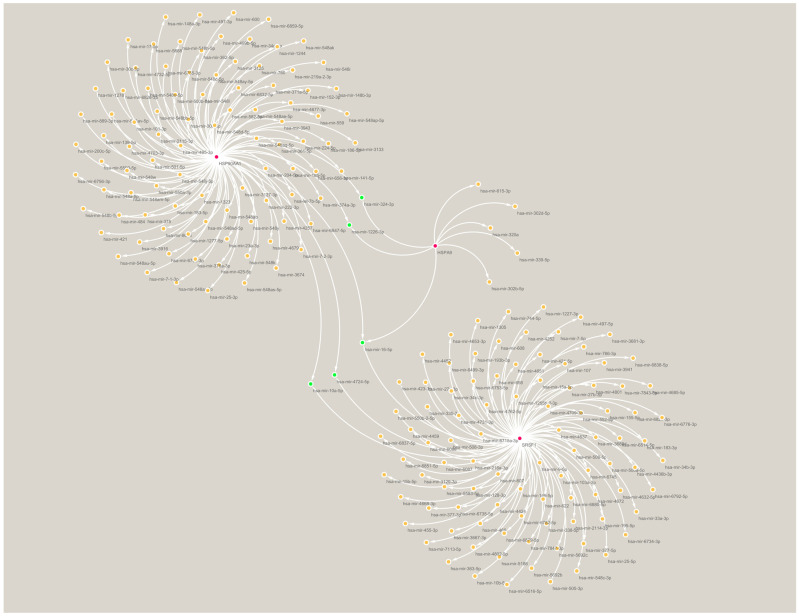
miRNA–mRNA regulatory network of the three hub genes. Red indicates hub genes, green indicates miRNAs associated with at least two of the hub genes.

We constructed a TF–mRNA regulatory network using ENCODE in the NetworkAnalyst tool (Figure 6). A total of 83 TFs were identified, and 11 of them were associated with only two of the pivotal genes: ZFX, ZNF584, ZNF24, POLR2A, FOXJ2, TAF7, THRAP3, MTA1, GTF2E2, MYC, and ZNF644. The only TF that was associated with all three pivotal genes was KLF9. We also constructed an mRNA–miRNA network using miRTarBase v8.0 in the NetworkAnalyst tool (Figure 7). A total of 200 miRNAs were identified, and four of them were associated with only two pivotal genes; namely, mir-324-3p, mir-1226-3p, mir-4724-5p, and mir-10a-5p. The only miRNA that was associated with all three hub genes was mir-16-5p. These TFs and miRNAs had high levels of interaction with the three hub genes in the network, indicating they may regulate these genes.

### 2.6. Drug Prediction

We used cMAP to scan the 81 common DEGs for drug-related signatures. A total of 2429 drugs and 171 cMAP classes were predicted. The highest score was for PI3K inhibitor (enrichment score 95.20) and the lowest score was for Tubulin inhibitor (enrichment score −99.48). We screened the top 10 drugs with negative correlations with the common DEGs, and found that 60% of them were Tubulin inhibitors (Table 2), and the highest ranked drugs were vindesine and ON-01910 (both scores −98.77).

### 2.7. Validation by Molecular Docking Simulations

**Figure 8:**
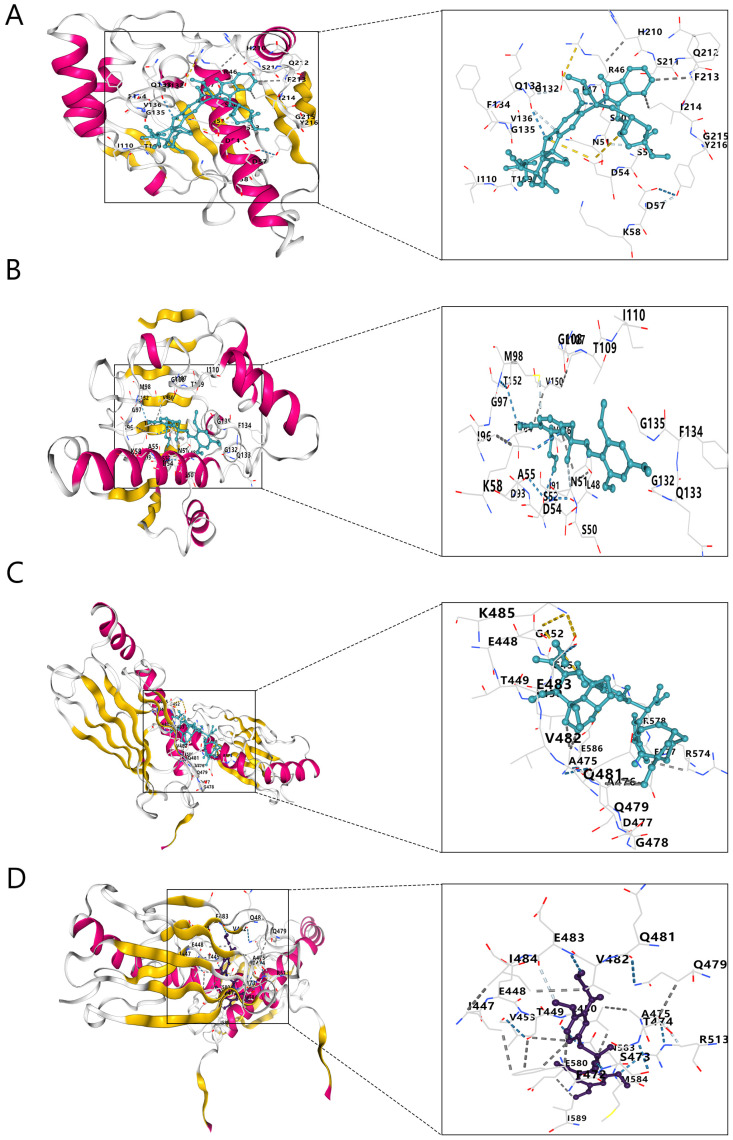
Molecular docking structures. (**A**) HSP90AA1 with vindesine. (**B**) HSP90AA1 with ON-01910. (**C**) HSPA9 with vindesine. (**D**) HSPA9 with ON-01910.

The structure files of vindesine and ON-01910, the drugs with the highest negative scores, were downloaded from PubChem for docking to the three pivotal genes. We used the 3D structure of ON-01910 and the 2D structure of vindesine because its 3D structure was not available in PubChem. The structure files of HSP90AA1, SRSF1, and HSPA9 were downloaded from the RCSB Protein Data Bank (PDB). The results show that vindesine and ON-01910 docked with HSP90AA1 and HSPA9 with binding fractions <−5 for all the complexes (Table 3 and Figure 8). However, for the docking of vindesine and ON-01910 with SRSF1, the ligand conversion failed and docking was not completed.

## 3. Discussion

Cardiovascular complications caused by COVID-19 have been the focus of much attention, including the pathogenesis of COVID-19 comorbidities of cardiovascular diseases, such as acute myocardial infarction and myocarditis [19,20]. Disease signature genes for COVID-19 or ICM have also been reported. However, little research has been focused on ICM and COVID-19 comorbidity [21,22,23,24,25]. We therefore explored potential biomarkers and molecular mechanisms of COVID-19 and ICM.

We first identified 81 common DEGs, 28 up-regulated and 53 down-regulated, between ICM and COVID-19. We hypothesized that these genes were associated with the potential mechanisms of the two co-morbidities. We then performed an enrichment analysis of the DEGs and discovered that the most enriched GO term under biological process was angiogenesis. Amir et al. [26] reviewed pathological angiogenesis caused by endothelial cell dysfunction in COVID-19, and Liao et al. [27] suggested that cell therapy could promote angiogenesis to improve ICM. We therefore speculated that angiogenesis may be one of the phenotypes of the co-morbidities.

To further explore the molecular mechanism of ICM and COVID-19 co-morbidity, we constructed a PPI network of the 81 common DEGs, and found that 62 of them interacted with each other. After screening and validation, we identified three pivotal genes (*HSP90AA1*, *HSPA9*, and *SRSF1*) that may be candidate biomarkers. The internal and external validation suggested that the three pivotal genes may have considerable diagnostic value, which will help further explore COVID-19 and ICM pathogenesis and diagnosis in future studies. These three genes have been shown previously to play crucial roles in angiogenesis.

HSP90AA1 (also known as HSP90A), a therapeutic target for cardiovascular disease and cardiac ageing, has been reported to mediate angiogenesis by direct binding to semi-synthetic triterpenoids [28,29], and it was found to be strongly related to the severity degree of COVID-19 disease [30]. HSP90AA1 was found to be a risk factor for the combination of cardiovascular disease and COVID-19 [31]. HSPA9 (also known as Mortalin) can promote angiogenesis and tumor progression by activating the Wnt/β-linked protein signaling pathway [32]. SRSF1 regulates differentiation, proliferation, and adaptive response of immune cells to various stimuli [33]. The YAP–RUNX2–SRSF1 axis has been reported to promote angiogenesis [34], and the circular RNA circSMARCA5 was found to regulate vascular endothelial growth factor A (VEGFA) angiogenesis by binding to SRSF1 [35]. These findings suggest that SRSF1 also has a close association with angiogenesis. Therefore, it is reasonable to speculate that angiogenesis is a key phenotype of ICM and COVID-19.

TFs and miRNAs often act together to regulate gene expression. To identify regulatory factors related to the pivotal genes, we constructed transcription factor and miRNA regulatory networks for these hub genes. One miRNA (hsa-mir-16-5p) and one TF (KLF9), which have been shown to be related to angiogenesis, were found to bind to all three hub genes. For example, knockdown of KLF9 inhibited retinal angiogenesis [36], and knockdown of miR-16-5p increased the level of vascular endothelial growth factor (VEGF) and increased cell viability and angiogenesis [37]. These findings provide insights that can be used to explore the molecular mechanisms underlying the co-pathogenesis of COVID-19 and ICM, and to determine whether hsa-mir-16-5p and KLF9 can regulate angiogenesis by regulating the pivotal genes throughout the course of the diseases.

Finally, drug prediction that targets key genes is feasible. For example, the inhibitor developed for the polymerase PARP-1 and HSPA9 can be used against COVID-19 [38]. We performed drug predictions using cMAP for the 81 common DEGs. To validate their binding effects to the pivotal genes, we selected the drugs with the highest negatively ranked binding scores for molecular docking with the three hub genes. The binding scores of vindesine and ON-01910 were <−5 for docking with HSP90AA1 and HSPA9, which suggested that these two drugs bound spontaneously to the proteins. However, the docking of these two drugs with SRSF1 suggested a ligand conversion error rather than their inability to dock. We considered that the SRSF1 protein structure that we used did not support the conversion of ligands; therefore, whether these drugs can bind to SRSF1 needs to be further investigated. Despite the failure of docking with SRSF1, the docking of vindesine and ON-01910 with HSP90AA1 and HSPA9 tentatively validated the feasibility of these drugs binding to the hub genes, indicating that vindesine and ON-01910 may be disease-specific agents of ICM and COVID-19.

In this study, common DEGs and potential biomarkers of ICM and COVID-19 were discovered for the first time. Our results will help to further clarify the pathophysiology of both these diseases. Our study has some shortcomings. We integrated publicly available sequencing and gene expression data for the analysis, and therefore batch differences in the integration of sequencing chips from different platforms cannot be ruled out. Although external RNA sequencing datasets were used for validation by molecular docking, this was performed only at the mRNA transcriptome level and only with computer simulations. In vivo and ex vivo experimental validation is needed to verify these findings in the future.

## 4. Materials and Methods

### 4.1. Preparation of Data

Gene expression data for many different disorders are publicly available in the GEO (www.ncbi.nlm.nih.gov/geo, accessed on 14 December 2022). GEO2R is a freely accessible internet tool for analyzing differentially expressed genes [39]. The GSE164805 dataset was generated using an Agilent-085982 Arraystar human lncRNA V5 microarray platform. The dataset contains 10 COVID-19 samples and 5 control samples (13 males and 2 females). For all the patients, the diagnosis was confirmed by reverse transcription-polymerase chain reaction (RT-PCR) for COVID-19 levels. Median age was 53.5 and 56 years for healthy individuals and patients with COVID-19, respectively. The GSE5406 dataset was generated using an Affymetrix human genome U133A array platform. The dataset contained 108 ICM samples and 16 control samples. All the patients had New York heart association class 3 to 4 symptoms and left ventricular systolic dysfunction, and all the controls had normal left ventricular function. The external datasets GSE211979 and GSE116250 were both generated by high-throughput sequencing on Illumina HiSeq2500 platforms. GSE211979 contains 16 COVID-19 and 5 control samples. All the patients were diagnosed by RT-PCR for COVID-19. The control samples, which were matched for age and sex, were from the Personalized Environmental and Genetic Study cohort. GSE116250 contains 13 ICM samples and 14 control samples (21 male and 6 female). The patients with ICM were identified based on medical history; all of them had significant obstructive coronary artery disease or myocardial infarction. The control group had no significant cardiac history and had ≥25% shortening of the left ventricular echocardiogram. Median age was 53.5 and 56 years for the control and ICM samples, respectively.

### 4.2. Collection of Common DEGs for COVID-19 and ICM

Differential gene analysis was performed using GEO2R. Statistical significance was defined as adjusted *p* value < 0.05. The screening thresholds to identify DEGs in the two internal datasets were log_2_(fold change) >0.5 (up-regulated DEGs) and log_2_(fold change) <−0.5 (down-regulated DEGs). The results were collated separately, including the removal of unannotated and/or duplicate genes. The Venn diagrams were integrated to identify DEGs co-regulated or down-regulated by COVID-19 and ICM. The data were visualized using Hiplot (ORG) (https://hiplot.org, accessed on 1 January 2023) [40].

### 4.3. Enrichment Analysis of Common DEGs for COVID-19 and ICM

Functional enrichment analyses were performed on the identified common DEGs using DAVID [41]. Statistical significance was set as *p* value < 0.05. Bubble plots were used to visualize the GO results, and a string plot was used to visualize the KEGG results.

### 4.4. Construction of PPI Networks to Obtain Hub Genes

Protein functional and regulatory interactions are a major source of complexity in cells. PPI network analysis can help to gain a deeper understanding of disease development. Common DEGs were uploaded to STRING (https://string-db.org, accessed on 5 January 2023) to construct a network of PPIs [42]. The networks were optimized using Cytoscape software 3.8.2 [43]. The top 10 genes in the networks were screened using four methods (MCC, EPC, Degree, BottleNeck) in the cytoHubba plugin to identify hub genes, which were then visualized in Venn diagrams. The expression data of the identified hub genes were integrated. After calculating the mean value for multiple gene probes that corresponded to the expression of the same hub gene, a correlation test was performed on the hub genes.

### 4.5. ROC Curve Validation

GSE5406 and GSE164805 were used as internal datasets, and GSE116250 (ICM) and GSE211979 (COVID-19) were used as external datasets. ROC curves were generated to assess the diagnostic efficacy of the hub genes for ICM and COVID-19.

### 4.6. Construction of TF–mRNA and mRNA–miRNA Regulatory Networks

MiRNAs (miRNAs) are small noncoding RNAs that bind to target mRNAs and regulate their expression by gene silencing or translational repression [44]. TFs also regulate gene expression both spatially and temporally [45]. The ENCODE and miRTarBase v8.0 databases in the NetworkAnalyst tool [46] (https://www.networkanalyst.ca/NetworkAnalyst, accessed on 12 January 2023) were used to build regulatory TF–mRNA and mRNA–miRNA networks for the hub genes, respectively. Cytoscape software was used to visualize the two networks.

### 4.7. Prediction of Potential Drugs of Common DEGs

CMap (https://clue.io/, accessed on 2 February 2023) is a database that connects genes, drugs, and disease states through common gene expression profiles [47]. The common COVID-19 and ICM co-expressed DEGs were uploaded to CMap to predict the drugs that may affect these genes. High absolute values of the score are the most relevant; “+” indicates that the trend of the drug effect is the same as the trend of the DEG, and “−” indicates the trend of the drug effect is opposite the trend of the DEG. Drugs with high negative correlation scores are considered the most relevant and can potentially down-regulate up-regulated genes and up-regulate down-regulated genes.

### 4.8. Molecular Docking Validation

Molecular docking is used to explore the binding properties of 3D ligand–receptor structures [48,49] and is widely used in drug discovery. The CB-DOCK2 software for molecular docking uses the tertiary structures of proteins and small ligand molecule. CB-DOCK2 has been used to validate the binding scores of key genes to small drug molecules [50,51]. The 3D structure files of the proteins encoded by the key hub genes were downloaded from RCSB PDB (pdb format) (https://www.rcsb.org/, accessed on 5 February 2023) [52]. The 3D structure files for the predicted drugs were downloaded from PubChem (sdf format) (https://pubchem.ncbi.nlm.nih.gov/, accessed on 5 February 2023). Molecular docking of the hub genes and drugs provide docking scores that indicate how well a drug binds to a protein; low scores indicate good binding.

## 5. Conclusions

In this study, *HSP90AA1*, *HSPA9*, and *SRSF1* were identified as markers for the co-pathogenesis of COVID-19 and ICM. We also found that the co-pathogenesis of these diseases may be related to angiogenesis. Vindesine and ON-01910 were predicted as potential therapeutic agents for COVID-19 and ICM. These biomarkers and the relationship of COVID-19 and ICM with angiogenesis may help to provide a deeper understanding of the comorbidity of ICM with COVID-19.

## Figures and Tables

**Table 1:** Top 10 proteins encoded by the hub genes in the PPI network ranked using four methods.

MCC			Degree			BottleNeck			EPC		
Rank	Name	Score	Rank	Name	Score	Rank	Name	Score	Rank	Name	Score
1	HSPA9	44	1	HSP90AA1	13	1	HSP90AA1	15	1	HSP90AA1	23.483
2	SRSF1	40	2	SERPINE1	7	2	SERPINE1	14	2	HSPA9	22.535
3	HSP90AA1	12	3	COL5A1	7	3	GJA1	13	3	ENO1	22.002
4	KLF9	3	4	ENO1	7	4	ITGA5	9	4	SRSF1	21.863
5	MTA1	2	5	HSPA9	7	5	HSPA9	7	5	SNRPB	21.297
5	TAF7	2	5	SRSF1	7	6	COL5A1	6	6	DDX39A	21.107
5	POLR2A	2	5	LUM	6	6	ENO1	6	7	DNAJB1	20.345
5	THRAP3	2	5	HNRNPU	6	6	SRSF1	6	8	SERPINE1	20.243
5	ZNF644	2	5	SNRPB	6	9	MAP1LC3B	5	9	HNRNPU	20.223
5	ZFX	2	5	DDX39A	5	9	SNRPB	5	10	GJA1	20.069

**Table 2:** Top 10 drugs with the highest negative correlations with the common DEGs predicted using cMAP.

Score	ID	Name	Description	Compound CID
−98.77	BRD-K59753975	vindesine	Tubulin inhibitor	40839
−98.77	BRD-K35687265	ON-01910	PLK inhibitor	73707390
−98.56	BRD-M30523314	vinorelbine	Tubulin inhibitor	73707424
−98.52	BRD-K82109576	vincristine	Tubulin inhibitor	5388992
−98.48	BRD-K91623615	ABT-751	Tubulin inhibitor	3035714
−98.43	BRD-K47869605	podophyllotoxin	Microtubule inhibitor	10607
−98.41	BRD-K52075715	oxibendazole	Tubulin inhibitor	4622
−98.31	BRD-K79131256	albendazole	Anthelmintic	2082
−98.2	BRD-K12539581	nocodazole	Tubulin inhibitor	4122
−98.17	BRD-K28120222	parthenolide	NFkB pathway inhibitor	7251185

**Table 3:** Docking of the three hub genes with predicted drug molecules.

Score	Vindesine	ON-01910
HSP90AA1	−8.1	−6.4
SRSF1	Receptor pdbqt transfer error.	Receptor pdbqt transfer error.
HSPA9	−7.9	−6.9

## Data Availability

The datasets analyzed in this study were acquired from the GEO database (https://www.ncbi.nlm.nih.gov/geo/, accessed on 14 December 2022).
